# Remote Tracking of Phase Changes in Cr_2_AlC Thin Films by *In-situ* Resistivity Measurements

**DOI:** 10.1038/s41598-019-44692-4

**Published:** 2019-06-04

**Authors:** Bastian Stelzer, Xiang Chen, Pascal Bliem, Marcus Hans, Bernhard Völker, Rajib Sahu, Christina Scheu, Daniel Primetzhofer, Jochen M. Schneider

**Affiliations:** 1 Materials Chemistry, RWTH Aachen University, D-52074 Aachen, Germany; 2 Max-Planck-Institut für Eisenforschung GmbH, D-40237 Düsseldorf, Germany; 3 Department of Physics and Astronomy, Uppsala University, S-75120 Uppsala, Sweden

**Keywords:** Surfaces, interfaces and thin films, Phase transitions and critical phenomena

## Abstract

Resistivity changes of magnetron sputtered, amorphous Cr_2_AlC thin films were measured during heating in vacuum. Based on correlative X-ray diffraction, *in-situ* and *ex-situ* selected area electron diffraction measurements and differential scanning calorimetry data from literature it is evident that the resistivity changes at 552 ± 4 and 585 ± 13 °C indicate the phase transitions from amorphous to a hexagonal disordered solid solution structure and from the latter to MAX phase, respectively. We have shown that phase changes in Cr_2_AlC thin films can be revealed by *in-situ* measurements of thermally induced resistivity changes.

## Introduction

Cr_2_AlC belongs to the M_n+1_AX_n_ phases, where M is a transition metal, A an A group element and X represents either C or N, a class of nanolaminates which have attracted considerable attention within the last decade due to their unusual combination of properties^1,2^. These characteristics are caused by the alternation of layers with metallic bonds in-between M and A elements and covalent/ionic bonds in the case of M-X layers^2–4^. Thus, MAX phases exhibit ceramic properties as for instance high stiffness^5,6^ as well as typically metallic features such as good machinability^3^ and high thermal and electrical conductivity^5,7–10^. MAX phases are compensated conductors exhibiting a metal-like behavior of the electrical resistivity *ρ* upon heating^2,7^. Density functional theory calculations show that the density of states at the Fermi level and thus the conductivity is dominated by the d states of the transition metal M^11^. The MAX phase Cr_2_AlC additionally exhibits excellent oxidation resistance due to the formation of a dense alumina scale^12–15^. The electrical resistivity of bulk Cr_2_AlC MAX phases has been studied at temperatures below room temperature^2,5,8^ as well as up to 900 °C^9,10^. Reported values of *ρ* at room temperature for Cr_2_AlC MAX phase range from *ρ* = 0.60 to 0.74 μΩ m^2,5,8–10^. Furthermore, it was shown that Cr_2_AlC is a self-healing material as cracks can be filled by selective oxidation of aluminum^16–20^. Thus, Cr_2_AlC may be of interest e.g. for nuclear applications^21,22^, heat exchangers^23^ or aero-engines^20^.

X-ray amorphous Cr_2_AlC powder samples synthesized by physical vapor deposition (PVD) have been analyzed by differential scanning calorimetry (DSC) by Walter *et al*.^24^ and Abdulkadhim *et al*.^25^. Based on correlative DSC and *ex-situ* X-ray powder diffraction (XRD) measurements Abdulkadhim *et al*.^25^ identified the formation of Cr_2_AlC MAX phase at 610 °C. Additionally, the presence of the disordered solid solution (Cr,Al)_2_C_x_ was observed at 560 °C. Thin film synthesis of this phase by sputter deposition was previously reported at substrate temperatures of 300 °C by Shtansky *et al*.^26^. *Ex-situ* annealing of the sample at 800 °C resulted in the formation of Cr_2_AlC MAX phase^26^. (Cr,Al)_2_C_x_ is structurally similar to the Cr_2_AlC MAX phase, which was suggested to consist of three perfectly ordered (Cr,Al)_2_C_x_ unit cells with a stacking sequence of Cr-Cr-Al-Cr-Cr-Al exhibiting a non-metal sub lattice order of C-vac-vac-C-vac-vac^25^.

It is the ambition of this work to detect phase changes by *in-situ* resistivity measurements during heat treatment. To this end, amorphous Cr-Al-C thin films are annealed to various temperatures up to 800 °C in a vacuum furnace, while *in-situ* resistivity measurements are performed. *Ex-situ* structural analysis reveals the formation of the disordered solid solution between 540 °C and 560 °C and a subsequent phase change to Cr_2_AlC MAX phase in the temperature range from 580 °C to 600 °C. Here it is shown that these structural transitions result in characteristic resistivity changes which enable remote tracking of phase changes by *in-situ* resistivity measurements.

## Methods

### Synthesis

Cr_2_AlC thin films were synthesized by direct current magnetron sputtering in an industrial chamber (CC800/8, CemeCon AG, Wuerselen, Germany). A Cr:Al:C compound target exhibiting a stoichiometry of 2:1:1 was employed (provided by PLANSEE Composite Materials GmbH, Lechbruck am See, Germany). The base pressure was below 0.1 mPa and the argon pressure during the deposition was set to 190 mPa. The target power density was 2.3 W cm^−2^. 10 × 10 × 0.5 mm single crystalline MgO substrates (Crystal GmbH, Berlin, Germany) were located at a distance of 5 cm to the plasma source and were kept at floating potential. No substrate heating was applied during deposition.

Thin films for *in-situ* heating transmission electron microscopy (TEM) measurements were deposited with the same deposition parameters on polycrystalline NaCl substrates. The deposition time was adjusted to grow an approx. 85 nm thick thin film. The as deposited film was separated from the NaCl substrate by dissolving in distilled water. The obtained film flakes were cleaned in distilled water, isopropanol and acetone prior to TEM analysis.

### Characterization

Electrical resistivity measurements during annealing experiments were performed in a vertical tube high vacuum furnace. 14 samples were heated to various temperatures in between 400 and 800 °C. No holding time was applied and a heating and cooling rate of 5 K min^−1^ was used. The base pressure was below 3∙10^−6^ mbar. The resistivity was measured *in-situ* employing a Van-Der-Pauw setup including a Keithley 2611B System SourceMeter using a current of 5 mA.

For determination of the chemical composition an as deposited sample was characterized by time-of-flight elastic recoil detection analysis (TOF-ERDA) and elastic backscattering spectroscopy (EBS) at the tandem accelerator laboratory of Uppsala University. For TOF-ERDA, ^127^I^8+^ projectiles with a primary energy of 36 MeV were employed. Further details can be found in reference^27^ and its supplements. EBS was carried out using 4.5 MeV ^4^He^+^ ions, a detection angle of 170° and thus employing the strong ^12^C(^4^He,^4^He)^12^C elastic resonance at ~4.260 MeV^28^.

Structural analysis by XRD was carried out in a Siemens D5000 system equipped with a Cu radiation source. The 2θ range from 10 to 90° was scanned in unlocked coupled setup with a tilt angle of 2° with respect to the substrate surface to avoid substrate peaks. The step size was set to 0.02° at a scan time of 10 s per step. Further structural characterization of the thin films was carried out using a Tecnai F20 TEM operated at a voltage of 200 kV. Cross-sectional TEM foils were prepared using a standard lift-out method in a DualBeam focused ion beam (FIB) system (Helios NanoLab 660). A thin layer of platinum was deposited on the film surface for protection against the beam damage.

Flakes obtained from NaCl substrates were positioned on a Nano-Chip (DENSsolutions). The *in-situ* heating experiment was conducted with a double tilt heating holder from DENSsolutions in a JEOL JSM 2200F field emission gun TEM. The selected area electron diffraction (SAED) patterns were obtained using an acceleration voltage of 200 kV. The sample was heated to a nominal temperature of 700 °C at a heating rate of 5 K min^−1^. Temperatures were obtained from the software, which is used to read out the chip. For a similar chip Niekiel *et al*. measured deviations between experimentally determined temperatures and the read out value of the software depending on the position of the sample on the chip^29^. The position of the window employed in this work corresponds to a temperature which is approx. 13 °C lower than the read out value. All temperatures obtained from the *in-situ* TEM heating experiments are corrected for this deviation.

## Results and Discussion

**Figure 1:**
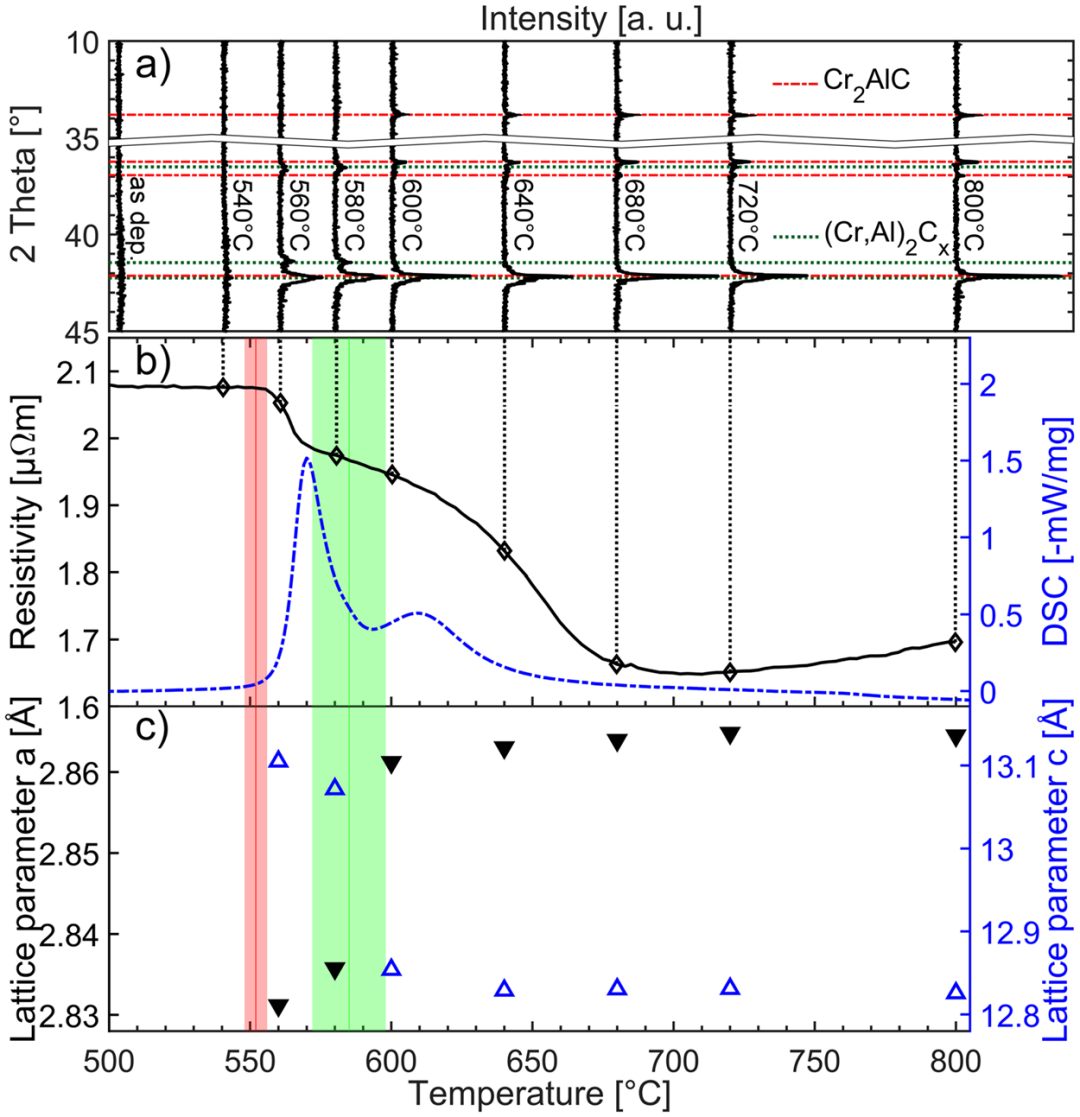
*In-situ* electrical resistivity measurements and correlative X-ray diffraction data (**a**) *Ex-situ* measured X-ray diffractograms of Cr_2_AlC samples annealed to various temperatures up to 800 °C indicated by dotted lines; (**b**) resistivity measured *in-situ* while heating (black) compared to DSC results by Abdulkadhim *et al*. (dashed)^25^. Red and green highlighted areas indicate average values and standard deviation of the onset of characteristic changes of resistivity for all measured samples; (**c**) lattice parameters determined from *ex-situ* XRD measurements. For better comparability of the c parameter the height of 3 stacked unit cells of (Cr,Al)_2_C_x_ is compared to a single unit cell of the MAX phase.

The diffractogram of the as deposited sample, see Fig. 1a, exhibits a wide hump around 42° indicating an X-ray amorphous thin film. The chemical composition of the as deposited thin film was measured by TOF-ERDA and EBS to be Cr: 49.9 ± 2.5 at.%, Al: 24.9 ± 1.2 at.%, C: 24.7 ± 1.2 at.% and O: 0.5 ± 0.3 at.%. Thus, the film is stoichiometric. The oxygen contamination is expected to stem from residual gas incorporation during the deposition process^30^. The venting temperature was lower than 50 °C to minimize modification of the surface composition by air exposure^31^.

The average film thickness was measured on 4 samples to be 3.8 ± 0.2 μm. Resistivity measurements at room temperature yielded an average of 2.15 ± 0.13 μΩ m for as deposited X-ray amorphous samples.

Figure 1b shows the result of one *in-situ* resistivity measurement during annealing from 500 °C to 800 °C. Up to a temperature of 550 °C, a negative temperature coefficient of resistance (TCR) *α*_*as dep, 300K*_ of −5.4 × 10^−5^ ± 0.7 × 10^−5^ K^−1^ was observed. This observation is in accordance with Mooij’s correlation predicting negative TCRs for amorphous transition metals with *ρ(0)* > 1.50 μΩ m and can be rationalized based on the mean free paths of electrons in the order of interatomic distances^32–34^.

At a temperature of 552 ± 4 °C a pronounced change in resistivity of 5.0 ± 0.7% within 20 °C was observed. As the temperature is increased to 585 ± 13 °C a second significant resistivity change of 3.0 ± 1.7% within 20 °C (17.0 ± 2.1% within 60 °C) is measured. The above given temperatures are averaged values and the corresponding temperature ranges represent the standard deviations stemming from 14 and 9 individual measurements for the first and second pronounced change of resistivity, respectively. For this purpose, the inflection point of the resistivity curve is employed as an indicator for the onset of the second pronounced change in resistivity. Above 700 °C the sample exhibited a positive TCR indicating a metal-like behavior of the temperature dependency as expected for MAX phases^2,10,35^.

**Figure 2:**
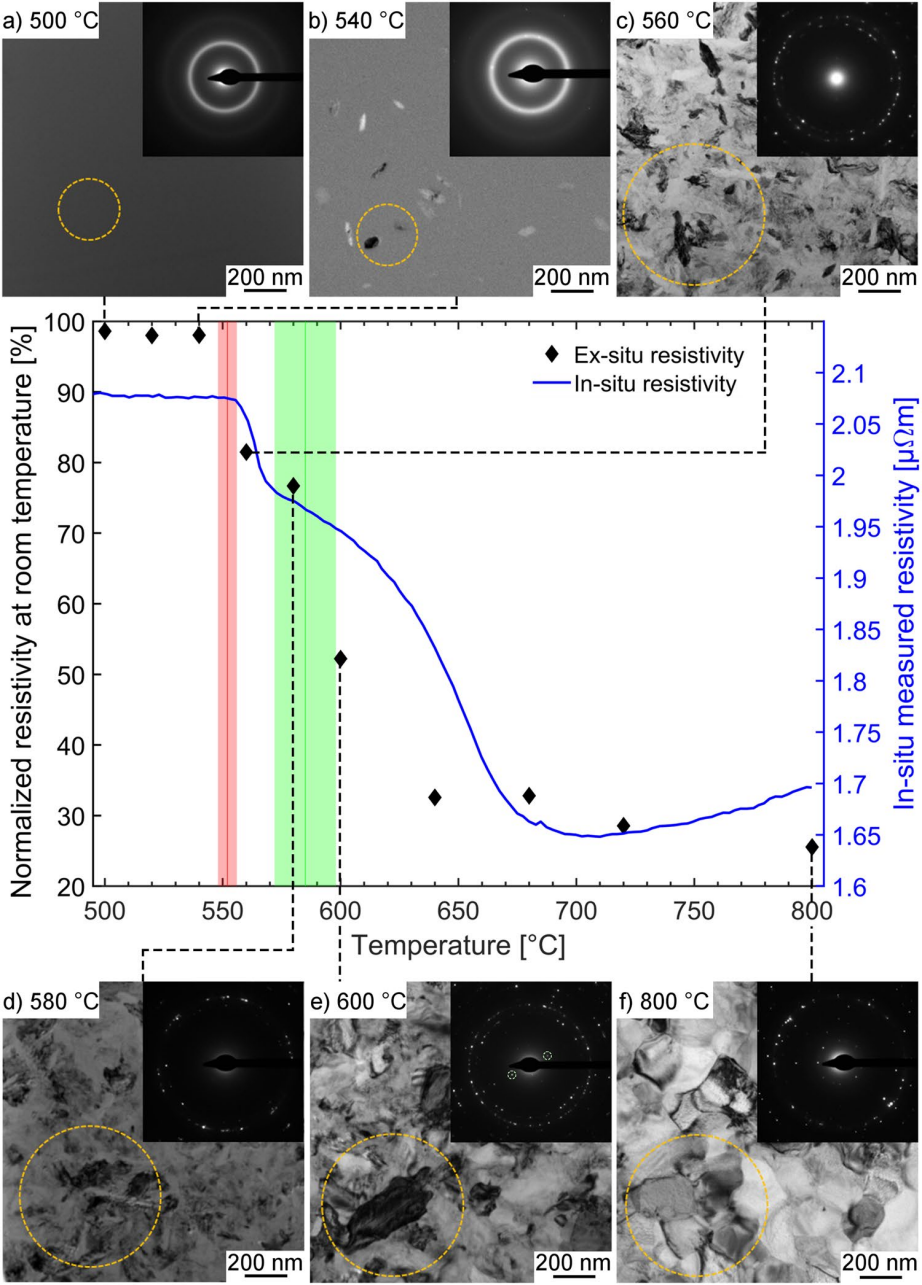
Microstructure and *ex-situ* SAED measurements compared to temperature dependence of resistivity (**a**–**f**): Bright field images and SAED patterns of FIB lamellae from Cr_2_AlC thin films after annealing at 500, 540, 560, 580, 600 and 800 °C in vacuum; Center: *In-situ* measured resistivity (blue) and *ex-situ* resistivity at room temperature (black) after annealing to various temperatures. The *ex-situ* resistivity data is normalized with respect to the initial resistivity at room temperature.

A similar trend was observed for the *ex-situ* resistivity measured at room temperature after annealing at temperatures of 500, 540, 560, 580, 600 and 800 °C as shown in Fig. 2. For an annealing temperature of ≤540 °C the measured resistivity at room temperature was reduced by less than 2% compared to the initial resistivity at room temperature. However, annealing to 560 °C resulted in a permanent change of resistivity by −18.5%, while heating a sample to 640 to 800 °C resulted in a resistivity of less than one third of the initial resistivity. Very good agreement between the temperature induced *in-situ* and *ex-situ* resistivity changes was obtained.

Structural changes due to annealing at different temperatures were probed by *ex-situ* XRD and are shown in Fig. 1a. A comparison of diffractograms of an as deposited thin film and one annealed to 540 °C does not reveal structural changes due to the heat treatment. Both diffractograms indicate the presence of X-ray amorphous thin films. Crystallization was observed for an annealing temperature of 560 °C which is consistent with Grieseler *et al*. reporting crystalline thin films after annealing Cr-Al-C multilayer systems at 550 °C^36^. At temperatures of 600 °C and higher an additional peak at 13.78° appears. At 560 to 580 °C a disordered solid solution (Cr,Al)_2_C_x_ is formed, which is consistent with Abdulkadhim *et al*.^25,26^. (Cr,Al)_2_C_x_ is structurally similar to the Cr_2_AlC MAX phase, which was suggested to consist of three perfectly ordered (Cr,Al)_2_C_x_ unit cells^25^. Due to the structural similarity between (Cr,Al)_2_C_x_ and Cr_2_AlC a positive phase identification of the MAX phase can be challenging. However, the (002) peak of Cr_2_AlC at 13.8° as well as the (101) peak at 36.90° are distinct indicators for MAX phase formation. Upon phase transformation the (006) peak of Cr_2_AlC MAX phase, which is structurally related to the (002) peak of (Cr,Al)_2_C_x_ at 41.35°, shifts to higher angles past the (103) MAX phase peak at 42.16°. Thus, samples annealed at 600 °C to 800 °C were identified as Cr_2_AlC MAX phase based on the presence of the (002), (006) and (101) peaks. Due to the peak overlap for both phases an unambiguous appraisal of the phase purity of the obtained MAX phase thin films based on diffraction data alone is not feasible.

With increasing annealing temperature, the lattice parameters change, see Fig. 1c. For a better comparability in-between MAX phase and disordered solid solution three stacked unit cells of (Cr,Al)_2_C_x_ are considered here. Thus, the actual *c* parameter of (Cr,Al)_2_C_x_ is one third of the here employed value. While the *a* lattice parameter increases with higher annealing temperatures from 2.831 to 2.865 Å, the *c* lattice parameter decreases from 13.105 to 12.826 Å, for temperatures of 560 °C and 800 °C, respectively. For the phase transition from disordered solid solution to the MAX phase between 580 and 600 °C relative changes in the lattice parameters of 0.90 and −1.66% are observed for *a* and *c*, respectively. Lattice parameters of both phases are in very good agreement with previously reported values^25^. However, both lattice parameters vary within the temperature region of the MAX phase, which may indicate higher crystal quality.

The XRD results are in agreement with *ex-situ* SAED measurements on lamellae extracted from annealed thin films by FIB as shown in Fig. 2. For the thin film which was annealed to 500 °C the SAED pattern exhibits only a diffuse ring indicating the presence of an amorphous structure and bright field images do not exhibit any features. However, small crystallites are already visible in the bright field image for the sample annealed to 540 °C. Due to the crystallite size and the small volume fraction of the crystalline material XRD is insensitive to this phase formation. Annealing to 560 °C induced a fully crystalline sample with elongated grains. However, only after annealing to 600 °C the (002) basal plane of the MAX phase structure was detected.

**Figure 3:**
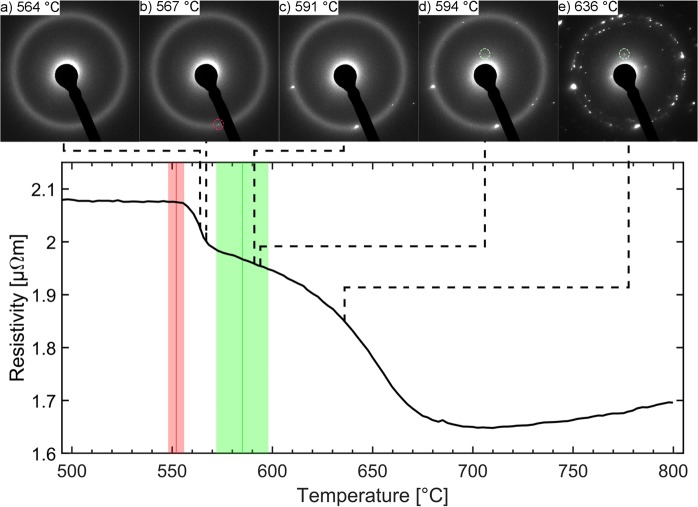
*In-situ* SAED measurements compared to temperature dependence of resistivity (**a**–**e**) SAED patterns obtained from *in-situ* heating TEM measurements at indicated temperatures; Bottom: *In-situ* measured resistivity. Red and green highlighted areas indicate average values and standard deviation of the onset of characteristic changes of resistivity for all measured samples. Highlighted diffraction signals are initial diffraction signals linked to (Cr,Al)_2_C_x_ disordered solid solution (red) and Cr_2_AlC MAX phase (green).

Both *ex-situ* diffraction techniques were applied to samples after heating and cooling. Therefore, compared to the *in-situ* experiment the annealing process was prolonged by the cool down procedure. This procedure may lead to further crystallization of the samples prior to analysis shifting the necessary temperature for observations to lower values. In an effort to narrow down the phase transition temperature range, *in-situ* heating TEM measurements were conducted. Representative SAED patterns are shown in Fig. 3. Up to a temperature of 564 °C (Fig. 3a) the SAED data is consistent with XRD measurements indicating the presence of amorphous Cr_2_AlC. At 567 °C a diffraction signal belonging to the (101) plane of (Cr,Al)_2_C_x_ was detected (Fig. 3b), whereas the first diffraction signal stemming from the (002) plane of the MAX phase was identified at 594 °C (Fig. 3d). With increasing temperature, the intensity of the diffraction signals stemming from crystalline material increased as the fraction of amorphous material was reduced (Fig. 3e). The phase transformation from (Cr,Al)_2_C_x_ to Cr_2_AlC MAX phase at 591 to 594 °C narrows down the transition temperature range of 580 to 600 °C obtained by *ex-situ* XRD and SAED analysis. However, the reported temperatures for the amorphous to (Cr,Al)_2_C_x_ transition at 564 to 567 °C are slightly above the temperature range from 540 to 560 °C identified by *ex-situ* analysis. This difference may on the one hand be caused by uncertainties associated with the temperature calibration as our appraisal is based on the assumption that the chip employed in this study shows identical behavior as the chip employed by Niekiel *et al*.^29^. On the other hand, the extended annealing time by the cooling procedure for the *ex-situ* analyzed samples and the smaller analyzed sample volume may also have influenced the observed transition temperatures. These results are supported by *in-situ* SAED measurements performed on a lamella extracted by FIB milling from an as deposited amorphous Cr-Al-C thin film which yield comparable results (not shown here).

Abdulkadhim *et al*. performed DSC measurements on amorphous Cr-Al-C powder at a heating rate of 10 K min^−1^ and observed an endothermic reaction corresponding to the presence of (Cr,Al)_2_C_x_ at 560 °C^25^, see Fig. 1b. They identified a second peak in the DSC signal at 610 °C and linked it to the phase formation of Cr_2_AlC MAX phase^25^. Further analysis of these DSC data reveals that the onset temperatures of the phase transformations from amorphous to the disordered solid solution (Cr,Al)_2_C_x_ as well as from disordered solid solution (Cr,Al)_2_C_x_ to Cr_2_AlC MAX phase occur at 560 °C and 585 °C, respectively.

The morphology of samples annealed to 500, 540, 560, 580, 600 and 800 °C were analyzed by *ex-situ* bright field images of prepared FIB lamellas depicted in Fig. 2. All three thin films exhibit dense microstructures. As shown above, the sample annealed to 500 °C is X-ray amorphous which agrees with the featureless homogeneous cross section with no indication of crystallization. For the samples annealed to 560 °C and above crystallization was observed. The sample heated up to 560 and 580 °C, both identified by SAED as (Cr,Al)_2_C_x_, exhibited randomly oriented elongated grains (Fig. 2c,d). After the transition to the MAX phase by annealing to 600 °C a trend towards larger and equiaxed grains was observed. Further annealing to 800 °C leads to further grain growth. This observation may explain the decrease in measured *ex-situ* resistivity for the MAX phase samples annealed to temperatures ≥600 °C (Fig. 2). The electrical resistivity at 300 K after annealing to 680, 720 and 800 °C was measured to be 0.78, 0.57 and 0.56 μΩ m, respectively. Reported values for the Cr_2_AlC MAX phase are *ρ*(300 K) = 0.60 μΩ m by Ying *et al*.^10^, *ρ*(300 K) = 0.74 μΩ m by Hettinger *et al*.^2,5^, *ρ*(RT) = 0.63 μΩ m by Zhou *et al*.^9^ and *ρ*(RT) = 0.71 μΩ m by Tian *et al*.^8^. Thus, the values measured within this work range from the upper to the lower end of the known resistivity range with lower values at higher annealing temperatures. As the temperature is increased further after MAX phase formation the crystal quality improves by coarsening as indicated by diffraction peaks with larger intensity and decreased full width at half-maximum (Fig. 1a) as well as the larger grains observable in the bright field images (Fig. 2). The improvement in crystal quality enables lower resistivity values due to a reduced defect density. Thus, it is reasonable to assume that the above discussed resistivity range reported in literature^2,5,8–10^ is caused by a synthesis induced variation in crystal quality.

Comparing the phase transition temperature ranges obtained by XRD, SAED and DSC^25^ with the measured temperature dependent resistivity signal reveals that the resistivity changes are characteristic for the here observed phase transformations. *Ex-situ* SAED data indicate that the onset of crystallization of (Cr,Al)_2_C_x_ occurs below 540 °C while at 560 °C a fully crystalline structure is observed. Thus, the formation of a fully crystalline sample correlates with the first pronounced resistivity decrease at 552 ± 4 °C. The second pronounced decrease in resistivity measured at 585 ± 13 °C indicates the phase transition from (Cr,Al)_2_C_x_ to Cr_2_AlC MAX phase, which was shown to take place in the temperature range from 580 to 600 °C by both *ex-situ* diffraction techniques, XRD and SAED. Results on powder samples analyzed by *in-situ* SAED experiments indicate phase transitions at temperatures ≤15 °C above the average temperatures determined by *in-situ* resistivity measurements. DSC measurements on powder samples^25^ are in very good agreement with transition temperature ranges obtained by *in-situ* resistivity measurements for both phase formations. Hence, the correlation of measured structural changes with the measured resistivity changes reveals that changes in resistivity are characteristic for the here observed phase transformations.

Based on the correlation of measured structural changes with the measured resistivity changes it is evident that changes in resistivity are characteristic for the here observed phase transformations in Cr_2_AlC. Thus, measuring resistivity is proposed as a powerful yet technically comparatively simple tool to track the onset and progress of phase transitions without destructive material characterization. It is conceivable that this method can be employed to monitor structural changes during application. E.g. it would allow the estimation of amorphization due to irradiation in materials employed in nuclear applications. The here communicated research strategy for tracking phase changes may be utilized in fundamental research as well as in technological applications where phase changes are expected due to exposure to harsh environments, such as nuclear reactors.

## Conclusions

Annealing experiments of magnetron sputtered amorphous Cr_2_AlC samples were performed in vacuum at temperatures up to 800 °C. *In-situ* resistivity measurements revealed two characteristic changes of resistivity which were observed at 552 ± 4 °C and 585 ± 13 °C. These are in excellent agreement with DSC measurement by Abdulkadhim *et al*.^25^ and correlate with phase changes from amorphous to hexagonal (Cr,Al)_2_C_X_ as well as (Cr,Al)_2_C_X_ to Cr_2_AlC MAX phase observed by XRD and *ex-situ* as well as *in-situ* SAED. The results clearly reveal that phase changes in Cr_2_AlC thin films can be tracked by non-destructive resistivity measurements. These findings are relevant for other materials systems provided that the different phases exhibit differences in resistivity.

## Data Availability

The data and samples analyzed during the current study are available from the corresponding author upon request.
